# Naïve prey exhibit reduced antipredator behavior and survivorship

**DOI:** 10.7717/peerj.665

**Published:** 2014-11-06

**Authors:** Charles W. Martin

**Affiliations:** Department of Oceanography and Coastal Sciences, Louisiana State University, Baton Rouge, LA, USA

**Keywords:** Predator-prey, Crayfish, Trophic, Chemical cues, Bass, Olfactory, Trophic, Predation, Naivety

## Abstract

Prey naiveté has been hypothesized to be one of the major driving forces behind population declines following the introduction of novel predators or release of inexperienced prey into predator rich environments. In these cases, naïve prey may lack sufficient antipredator behavior and, as a result, suffer increased mortality. Despite this, some evidence suggests that many prey utilize a generalized response to predators. Here, the naiveté hypothesis is tested using a predator–prey pair sharing an evolutionary history: the red swamp crayfish (*Procambarus clarkii* Girard, 1852) and largemouth bass (*Micropterus salmoides* Lacépède, 1802). Using farm-reared, naïve crayfish and wild-caught, experienced individuals, laboratory experiments demonstrated that naïve, farmed crayfish lack behavioral responses to chemical cues from bass, both in terms of movement and use of structural refuge. In contrast, experienced crayfish responded strongly to the same cues. In a subsequent field tethering experiment, these naïve individuals suffered a three-fold increase in predation rate. Based on these results, recognition of predators may not be innate in all prey, and previous experience and learning likely play a key role in the development of antipredator behavior.

## Introduction

The ability of prey to recognize and respond to the threat of predation is critical to the survival and persistence of prey populations (Lima, 1998a; Lima, 1998b). Recent evidence has indicated that the fear of predation can have wide-ranging effects throughout the ecosystem and even exceed impacts to prey populations caused by consumption (Werner & Peacor, 2003; Preisser, Bolnick & Benard, 2005; Schmitz et al., 2008; Sih et al., 2010). As such, understanding how these non-consumptive interactions among predators and prey are exhibited is an important consideration when unraveling the complex nature of many food webs.

Prey utilize a variety of information to make decisions regarding the immediacy of threats, sources that include visual cues, olfaction, social signals, and other specialized sensory mechanisms (Martin et al., 2010). Typical responses of prey to the threat of predation include altering habitat preference patterns, foraging intensities, and/or life history strategies, all in an effort to decrease detection and capture by predators (Lima & Dill, 1990; Dill, Heithaus & Walters, 2003; Werner & Peacor, 2003). Prey that fail to recognize and respond to predators experience enhanced predation (Mathis & Smith, 1993a), a phenomenon that has been referred to as prey naiveté (Kovalenko et al., 2010).

Prey naiveté is often attributed either to: (1) the prey’s lack of exposure to a specific predator co-occurring over a geographic range, or (2) the absence of evolutionary history between predators and prey (Diamond & Case, 1986; Cox & Lima, 2006; Smith et al., 2008; Sih et al., 2010). The introduction of invasive predators (Sax & Gaines, 2008) serves as a powerful example of this latter form of naiveté. Dramatic declines in prey populations have been demonstrated following introduction of predators such as Nile perch (*Lates niloticus*), peacock bass (*Cichla* spp.), and Indo-Pacific lionfish (*Pterois volitans/miles*) (Zaret & Paine, 1973; Ogutu-Ohwayo, 1990; Albins & Hixon, 2008). Prey that share evolutionary history with a specific predator, however, are generally considered more resilient to predators by possessing some evolved antipredator behavior as a consequence of the evolutionary ‘arms race’ between predators and prey. Still, examples exist from a variety of hatchery-reared, inexperienced fishes lacking appropriate antipredator responses, including rainbow (*Oncorhynchus mykiss*) and brown trout (*Salmo trutta*), coho (*O. kisutch*) and Atlantic salmon (*S. salar*), and red drum (*Sciaenops ocellatus*) (Olla & Davis, 1989; Brown & Smith, 1998; Alvarez & Nicieza, 2003; Brown, Davidson & Laland, 2003; Stunz & Minello, 2001). This behavioral deficit may be attributed to hatchery selection and differential experience and likely incorporates other facets of the organism’s behavior in addition to predator recognition (foraging, habitat use, etc.). In fact, it has been hypothesized that hatchery-reared individuals used in restocking efforts rarely survive to adulthood (McNeil, 1991).

To date, conflicting evidence exists regarding prey’s capacity to innately recognize the threat of predation. Despite the pervasive effects of some invasive predators, other studies have indicated that some prey may have the ability to develop a response to introduced predators (Mathis & Smith, 1993b; Shave, Townsend & Crowl, 1994; Kovalenko et al., 2010; Chivers & Ferrari, 2013). As a result, the contrasting roles of learned behavior and naiveté are unclear and often confounded.

Crayfish are known to vary widely in their response to predators (Hazlett & Schoolmaster, 1998), with some species (*Pacifastacus leniusculus,* Blake and Hart 1993; *Paranephrops zealandicus*, Shave, Townsend & Crowl, 1994) exhibiting strong responses to chemical cues exuded from predators, and others (*Orconectes propinquus* and *O. virilus*, Willman et al., 1994) showing little response. Previous research (Gherardi et al., 2011) on exotic populations of red swamp crayfish (*Procambarus clarkii* Girard, 1852) has indicated that these organisms contain very complex responses to predators, including a generalized response to fish (a trait that likely contributes to its effectiveness as an invader) as well as the capacity to learn different levels of risk involved with specific predator chemical odors (Acquistapace, Hazlett & Gherardi, 2003). Here, a series of experiments are conducted on *P. clarkii* within its native range to test whether: (1) naïve individuals exhibit reduced antipredator responses, and (2) whether these predicted differences in behavior translate to higher mortality in field settings.

## Methods

### Study organisms

*Procambarus clarkii* are common inhabitants of freshwater and brackish environments in the southern United States, where they are important omnivores and key trophic intermediates (Gherardi, 2006). In Louisiana, crayfish aquaculture is a lucrative industry, with many rice farms modifying operations to accommodate a dual crop rotation with crayfish, which now yield greater value than rice crops (McClain & Romaire, 2007). Potential fish predators (such as bass and numerous species of sunfish) are removed from farms by draining ponds and filtering water upon refilling (McClain & Romaire, 2004). As such, farm-raised crayfish have been raised in environments free from fish predators and, as a result, present an opportunity to test the naiveté hypothesis.

Crayfish used in these experiments were sourced from five different commercial suppliers near Baton Rouge, LA (USA) to reduce the chance that any detected effect would be population-specific. For comparison, wild-caught crayfish from the Atchafalaya Basin, LA (USA), an area with many potential fish predators, were used. No significant difference was detected in size (mean cephalothorax length, 46.6 ± 4.30 mm) of individuals used in experiments (two-sample *t*-test: structure experiment *t*_66_ = 0.74, *p* = 0.462; movement experiment *t*_45_ = −0.18, *p* = 0.856; tethering experiment *t*_41_ = −0.15, *p* = 0.883). Largemouth bass (*Micropterus salmoides* Lacépède, 1802), an abundant predatory fish found throughout much of the United States, was used as the predator in laboratory trials (bass total length: 26–32 cm). Odor from one of five different bass was used and randomly assigned in trials. Bass were fed a diet of mosquitofish (*Gambusia affinis*) every other day while in captivity. All organisms used in experiments were housed for a minimal amount of time (<1 month) in captivity, on a 12:12 light:dark schedule, and complied with collection and ethics laws for the state of Louisiana (permit # 2580) and Louisiana State University (IACUC approval #14057), and released to the wild upon completion of experiments.

### Behavioral response to predators

The response of crayfish to bass was measured in two laboratory experiments. All trials were conducted in 4-L (23.3 cm × 15.5 cm × 16.5 cm) acrylic aquaria containing approximately 2 cm of sand sediment. Consistent environmental conditions (temperature = 22 °C, pH = 8.2) were maintained throughout trials and all tanks contained dechlorinated tap water and an airstone in the center of the tank. All crayfish and bass were starved for 24 h and crayfish were allowed to acclimate for 1 h prior to start of trials. No crayfish was used more than once in trials and aquaria were drained and rinsed between trials. Predator cues were introduced into trial tanks containing crayfish by pouring 50 mL of water taken from a 38-L tank (50.8 cm × 27.9 cm × 33.0 cm) containing a largemouth bass for 24 h. Additionally, trials were conducted using water from an identical tank containing no predator as a control. All trials were recorded using a high definition video camera for a period of 10 min to avoid breakdown of odors or acclimation to cues. Treatments for both of the following experiments were assigned randomly and included all possible combinations of two treatments: crayfish [2 levels: naïve (farmed) and experienced (wild)] and predator (2 levels: with predator scent and without).

Prey use structural refuges as deterrents to foraging predators (Heck & Thoman, 1981). In the first experiment (hereafter referred to as “structure experiment”), a 10 cm section of 4 cm diameter PVC pipe was placed in tanks as structure prior to adding crayfish. While this refuge is not natural, neither wild nor farmed crayfish had prior experience with this type of structure. The amount of time crayfish spent hiding in structure over the trial duration was tallied and trials were replicated 18 times for each unique treatment.

Another common response to predators is to decrease movement, thereby reducing the chances of detection by predators (Sih, 1987; Lima & Dill, 1990; Lima, 1998a). In fact, an estimated 60% of studies have documented this to occur across numerous and diverse taxa (Lima, 1998b). In a second experiment (hereafter referred to as “movement experiment”), the tank wall facing the camera was gridded off into 3 cm × 3 cm sections and the number of squares occupied by the head of the crayfish was tallied over the trial period. Cue-containing water was introduced using the same methods as the first experiment and trials were replicated 12 times for each unique treatment.

### Survivorship

To determine whether naiveté influences crayfish survival, a field tethering experiment was conducted in the Louisiana State University Lakes (30°24′54.03″N, 91°9′56.70″W) located near the campus of Louisiana State University in Baton Rouge, LA. While the presence and density of predators in lakes were not measured in this study, all of the lakes used in this study are known to contain numerous fish predators, many of which are targeted by recreational fishermen (Balkom, 2013), as well as other avian and mammalian predators. This experiment was conducted along the shorelines of five lakes in the system separated by roads (*n* = 5): University Lake, College Lake, Campus Lake, Lake Crest, and City Park Lake. All tether locations contained similar depth (<0.5 m) and water clarity (secchi depth ∼10 cm).

Crayfish were tethered by tying a knot in a 1 m section of monofilament fishing line (Berkely^®^ Trilene, 0.23 mm diameter) around the carapace of the crayfish. A drop of cyanoacrylate cement was used to hold the knot in place (Puntila, Martin & Valentine, 2012). The other end of the tether was tied to a labeled stake that was placed at the water’s edge. Five naïve and five experienced crayfish were tethered and placed at alternating locations along the banks at each of the five lakes. After 24 h, tethers were checked and considered consumed if missing (all stakes were accounted for after the trial duration). In addition, five individuals were tethered to a stationary object and kept in the lab, and no handling mortality or escape from tethers were detected over the trial period. While crayfish held in the lab had no stimuli from predators and, as a result, likely displayed fewer tail-flip escape behaviors that may have allowed them to escape tethers, this method has been used successfully in other crayfish studies (Garvey, Stein & Thomas, 1994; Englund & Krupa, 2000).

### Statistical analyses

Normality and homogeneity of variance were tested prior to analyses, and data were transformed if assumptions were not satisfied. The behavioral response to predators was analyzed using two-way ANOVAs with crayfish (farmed, wild) and predator (present, absent) and the interaction as factors. Response variables in these experiments were the proportion of time spent in structure (log(*x* + 1) transformed) for the structure experiment and number of squares occupied (square root transformed) in the movement experiment. When significant differences were detected, pairwise comparisons were made using Tukey’s post hoc test. Survivorship proportions (arcsine square root transformed) were analyzed using a one sample *t*-test to test whether the difference in survivorship between naïve and experienced crayfish was statistically different from zero (Peterson & Renaud, 1989; Pennings et al., 1998; Martin, Valentine & Valentine, 2010). All results were considered significant at *p* ≤ 0.05.

## Results

### Behavioral response to predators

**Figure 1:**
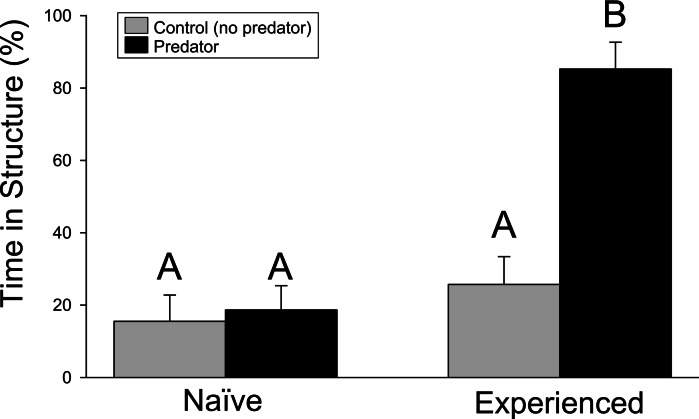
Structure experiment. The percentage (+ 1SE) of the trial period spent hiding in refuge. Different letters indicate statistically significant differences.

Crayfish responded strongly to water containing chemical cues from largemouth bass. However, the antipredator response from wild-caught, experienced crayfish significantly exceeded that of naïve, farmed individuals. In the structure experiment, significant differences were found for the crayfish treatment (*F*_1,68_ = 25.23, *p* ≤ 0.001), predator treatment (*F*_1,68_ = 16.30, *p* ≤ 0.001), and interaction term (*F*_1,68_ = 12.13, *p* = 0.001). Experienced crayfish exposed to predator cues spent approximately 80% of time hiding in the structure of the PVC pipe, more than any other treatment (Fig. 1). Pairwise comparisons indicated the significant difference was driven by the difference between experienced crayfish with predator cues and the other treatments (*p* ≤ 0.001 in all cases), while no difference was detected among the other treatments (experienced crayfish/control cues vs. naïve/control cues, *p* = 0.6970; experienced crayfish/control cues vs. naïve/predator cues, *p* = 0.8977; naïve/control cues vs. naïve/predator cues, *p* = 0.9794).

**Figure 2:**
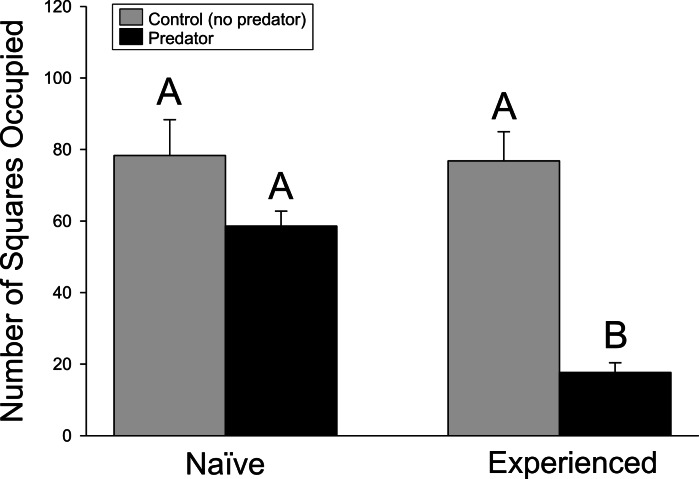
Movement experiment. The amount of crayfish movement, as indicated by the cumulative number of squares (+ 1SE) occupied over the trial period. Different letters indicate statistically significant differences.

Similar results were found in the movement experiment (Fig. 2), with significant differences detected in movement patterns for crayfish treatment (*F*_1,44_ = 18.18, *p* ≤ 0.001), predator treatment (*F*_1,44_ = 44.94, *p* ≤ 0.001), and interaction term (*F*_1,44_ = 17.62, *p* ≤ 0.001). Again, experienced crayfish with predators responded strongly to predator cues, moving less than all other treatments (*p* ≤ 0.001 in all cases), while other treatments did not significantly differ (experienced crayfish/control cues vs. naïve/control cues, *p* = 1.000; experienced crayfish/control cues vs. naïve/predator cues, *p* = 0.3232; naïve/control cues vs. naïve/predator cues, *p* = 0.3004).

### Survivorship

**Figure 3:**
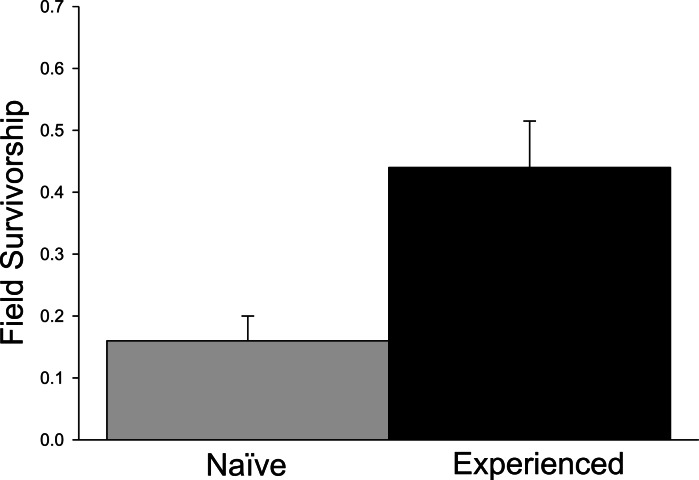
Tethering experiment. Proportion of surviving tethered crayfish after 24 h (+ 1SE).

Significant differences in field survivorship were also detected (Fig. 3; *t*_5_ = 10.20, *p* = 0.001), with experienced crayfish exhibiting increased survival. Survival of tethered experienced crayfish was approximately three times greater than that of tethered farmed crayfish over the 24 h trial period, with approximately 15% of naïve crayfish surviving compared to 45% of experienced crayfish.

## Discussion

The results of this study indicate that antipredator behavior in naïve crayfish is not elicited by bass predator odor and, presumably as a result, these naïve individuals exhibit decreased survivorship in field settings. Experienced crayfish, on the other hand, responded strongly to water containing chemical cues from the largemouth bass predator by spending more time in structural refuge and decreasing movement to avoid detection, both metrics previously used as indicators of predation threat (Lima & Dill, 1990). This work agrees with Acquistapace, Daniels & Gherardi (2004), who reported that aquacultured *P. clarkii* did not respond to alarm signals of injured conspecifics, in stark contrast to wild individuals (Hazlett et al., 2003). As a result, tethered experienced crayfish were much more successful in predator-rich environments than their naïve counterparts. It should be noted that, while bass are present in the field environments where crayfish were tethered, so too are a number of other predatory fishes, birds, reptiles, and mammals and naïve crayfish likely possessed little, if any, experience with these predators as well.

Some organisms are known to demonstrate antipredator responses to novel predators by identifying characteristics of the predator with similar features of a known predator. In this way, prey may “generalize” the threat of predation based on prior experiences (Ferrari, Messier & Chivers, 2007; Chivers & Ferrari, 2013). For example, Griffin, Evans & Blumstein (2001) conditioned wallabies to fox predators and found they respond similarly to an unknown predator sharing similar facial features (cats, *Felis catus*), but not to similarly sized organisms with different cranial morphology (goats, *Capra hircus*). It is possible that this applies to the olfactory cues used by *P. clarkii* as well, and exclusion of all fishes from crayfish farms in this study may have led to the lack of generalized response found in Gherardi et al. (2011), rather than simply a species-specific response to bass.

Naïve prey may suffer increased consumption as a result of the lack of predator recognition. Numerous examples illustrate the direct link between an organism’s antipredator behavior and trophic dynamics. For example, the invasion of non-native species can result in the presence of novel consumers that are unrecognized by prey (Cox & Lima, 2006; Sih et al., 2010), and this may be a contributing factor in the recent observation that invasive predators result in greater extinction rates than invasive plants (Sax & Gaines, 2008). Hatchery-reared fishes used in restocking efforts rarely survive to adulthood (McNeil, 1991, but see Brown, Ferrari & Chivers, 2013), presumably due to the lack of behavioral responses to predators (Stunz & Minello, 2001). Despite this, the manipulation used here (aquacultured versus wild crayfish) may include additional varying factors rather than simply the lack of predators in farms. For example, the handling time, diet, and overall health (though no specific differences were noted) all could vary between farmed and wild organisms. Moreover, the number of generations these farmed organisms are removed from the wild is unknown and may be an important covariate and thus requires additional study.

In some cases, organisms under strong predation pressure rapidly develop antipredator behavior (Kovacs et al., 2012; Anson & Dickman, 2013). The role of learning is well documented in aquatic organisms (Brown & Chivers, 2006; Ferrari, Wisenden & Chivers, 2010). Mathis & Smith (1993b) exposed fathead minnows (*Pimephales promelas*) to chemical stimuli of unknown pike (*Esox lucius*) predators, and when combined with cues of minnows in the predator’s diet, recognized dangerous situations with these characteristics. Similarly, Ferrari & Chivers (2006) conditioned fathead minnows to brook charr (*Salvelinus fontinalis*) predators and conspecific alarm cues at different intensities and found that minnows associated risk based on the highest cue concentration used during conditioning. Olla & Davis (1989) exposed naïve, hatchery raised coho salmon to lingcod predators and found that survivors were better able to survive future predation attempts. Previous studies have indicated this learned response can last in the order of weeks without further reinforcing exposure to the predator (Brown & Smith, 1998; Hazlett, Acquistapace & Gherardi, 2002; Lima, 1998b; Chivers & Ferrari, 2013).

Given the successful invasion of crayfish such as *P. clarkii* and *O. rusticus* throughout the world, it may be predicted based on results presented here that they would initially have very high mortality to predators. Hence, their survival in novel areas may depend on quickly learning potential predators. Indeed, previous studies have suggested that invasive *O. rusticus* and *O. virilis* crayfish need experience with predator odor to recognize it as a danger signal (Hazlett & Schoolmaster, 1998), yet may demonstrate a response to predator cues after only a two hour “training period” in which they were exposed to fish and alarm odors (Acquistapace, Hazlett & Gherardi, 2003). Hazlett, Acquistapace & Gherardi (2002) demonstrated that successful invasive crayfish species (including *P. clarkii*) have the capacity to remember cues from predators, combined with scent from crushed conspecifics, longer than other species of crayfish. Gherardi et al. (2011) exposed *P. clarkii* to known and unknown predator odors, including largemouth bass, and found that behavioral response is stronger when exposed to conspecific alarm odors than to fish odors, indicating crayfish also rely on predation of conspecifics as an indication of predation threats, especially when in conjunction with predator odors (Hazlett & Schoolmaster, 1998; Hazlett, Acquistapace & Gherardi, 2002; Acquistapace, Hazlett & Gherardi, 2003).

Our knowledge of the role of prior experience in determining prey antipredator behavior and the ensuing alteration to food webs is crucial given the increasing redistribution of organisms, both predators and prey, throughout the world. Results presented here suggest that naiveté is an important variable that influences the interaction between novel organisms and their predators, but it is also possible that learning and prior experiences play a strong role in structuring antipredator response, especially for successful invaders such as *P. clarkii* crayfish (Gherardi, 2006). Additional study across a broad range of taxa is required to develop more generalities regarding susceptibility of naïve prey to predators, including how spatial and temporal variability in the frequency and intensity of predation attempts shape prey behavior. Such research is necessary to understand the complexity of consumptive and nonconsumptive effects, and how these two forces influence food web functioning in an anthropogenically restructured world.

## Supplemental Information

10.7717/peerj.665/supp-1 Supplemental Information 1 Dataset for the movement experiment Dataset for the movement experiment.

10.7717/peerj.665/supp-2 Supplemental Information 2 Dataset for the refuge use experiment Dataset for the refuge use experiment.

10.7717/peerj.665/supp-3 Supplemental Information 3 Dataset for field tethering experiment Dataset for field tethering experiment.

## Additional Information and Declarations

### Field Study Permissions

The following information was supplied relating to field study approvals (i.e., approving body and any reference numbers): State of Louisiana Department of Wildlife and Fisheries: Permit #2580.
