# Supplementary material for: Naïve prey exhibit reduced antipredator behavior and survivorship
Source: PeerJ. 2014 Nov 6;2:e665. doi: 10.7717/peerj.665 (PMC4226725; doi:10.7717/peerj.665)
Supplement: Supplemental Information 1 — Dataset for the movement experiment. [file peerj-02-665-s001.pdf]

Crayfish Treatment	Predator Treatment	Squares Occupied During the Trial Period
farm	control	47
farm	control	48
farm	control	85
farm	control	147
farm	control	44
farm	control	97
farm	control	107
farm	control	54
farm	control	54
farm	control	42
farm	control	107
farm	control	108
farm	predator	54
farm	predator	48
farm	predator	94
farm	predator	74
farm	predator	64
farm	predator	65
farm	predator	49
farm	predator	42
farm	predator	62
farm	predator	48
farm	predator	54
farm	predator	49
wild	control	125
wild	control	100
wild	control	80
wild	control	87
wild	control	74
wild	control	40
wild	control	121
wild	control	79
wild	control	47
wild	control	57
wild	control	69
wild	control	43
wild	predator	9
wild	predator	18
wild	predator	15
wild	predator	35
wild	predator	29
wild	predator	7
wild	predator	13
wild	predator	8

wild	predator	25
wild	predator	7
wild	predator	26
wild	predator	20
