# Supplementary material for: Naïve prey exhibit reduced antipredator behavior and survivorship
Source: PeerJ. 2014 Nov 6;2:e665. doi: 10.7717/peerj.665 (PMC4226725; doi:10.7717/peerj.665)
Supplement: Supplemental Information 2 — Dataset for the refuge use experiment. [file peerj-02-665-s002.pdf]

Crayfish Treatment	Predator Treatment	Proportion of Time Spent in Structure
farm	control	0.000
farm	control	0.000
farm	control	0.000
farm	control	0.000
farm	control	0.567
farm	control	0.000
farm	control	0.018
farm	control	0.058
farm	control	0.000
farm	control	0.000
farm	control	0.000
farm	control	0.000
farm	control	0.000
farm	control	0.000
farm	control	0.000
farm	control	0.977
farm	control	0.785
farm	control	0.390
farm	predator	0.000
farm	predator	0.753
farm	predator	0.000
farm	predator	0.493
farm	predator	0.715
farm	predator	0.000
farm	predator	0.000
farm	predator	0.000
farm	predator	0.000
farm	predator	0.000
farm	predator	0.000
farm	predator	0.000
farm	predator	0.353
farm	predator	0.455
farm	predator	0.000
farm	predator	0.000
farm	predator	0.592
farm	predator	0.000
wild	control	0.000
wild	control	0.950
wild	control	0.000
wild	control	0.000
wild	control	0.683
wild	control	0.000
wild	control	0.487
wild	control	0.493
wild	control	0.025

wild	control	0.000
wild	control	0.000
wild	control	0.750
wild	control	0.000
wild	control	0.467
wild	control	0.200
wild	control	0.000
wild	control	0.570
wild	control	0.000
wild	predator	1.000
wild	predator	1.000
wild	predator	1.000
wild	predator	1.000
wild	predator	1.000
wild	predator	0.158
wild	predator	1.000
wild	predator	1.000
wild	predator	0.800
wild	predator	1.000
wild	predator	0.430
wild	predator	1.000
wild	predator	1.000
wild	predator	1.000
wild	predator	0.957
wild	predator	1.000
wild	predator	1.000
wild	predator	0.000
