# Supplementary material for: Naïve prey exhibit reduced antipredator behavior and survivorship
Source: PeerJ. 2014 Nov 6;2:e665. doi: 10.7717/peerj.665 (PMC4226725; doi:10.7717/peerj.665)
Supplement: Supplemental Information 3 — Dataset for field tethering experiment. [file peerj-02-665-s003.pdf]

	Proportion of Wild Crayfish Surviving	Proportion of Farmed Crayfish Surviving
site 1	0.6	0.2
site 2	0.4	0.2
site 3	0.2	0.0
site 4	0.6	0.2
site 5	0.4	0.2
